# The Central Hospitals Board Question

**Published:** 1896-05-02

**Authors:** 


### The Hospital

AN INSTITUTIONAL JOURNAL OF

The Medical Sciences and Hospital Administration,

WITH

AN EXTRA NURSING SUPPLEMENT.

VOL. XX.—No. 501.] [MAY 2, 1896.

### THE CENTRAL HOSPITALS BOARD QUESTION.

ONCE more the proposal, originating in the Lords' Committee on the metropolitan hospitals, to establish a Central Hospitals Board has been brought into prominence by a meeting called by the Charity Organisation Society on April 13th, and by a conference of hospital authorities held on April 23rd under the auspices of the Hospitals Association. Seeing that the scheme formulated by the Charity Organisation Society would create a board of 167 members, and that the main source to which it looks for funds are the trustees of the parochial charities, whose available income is, we are informed, already smaller than they require for the work entrusted to them under their Act of Parliament, this scheme as it stands must be regarded as impracticable. Those who are interested in the question would do well, however, to compare the proposals embodied in the scheme of the Charity Organisation Society—copies of which may be obtained on application to Mr. Loch—with those formulated by the Lords' Committee and by the special committee appointed at a conference held at Spencer House on July 22nd, 1892. There is also a further scheme prepared by Sir Douglas Galton's committee, Sir Douglas Galton having been the means of rejecting the proposals formulated by the Spencer House Special Committee. We have no space to summarise these various schemes, but they will be found set forth at length in Burdett's "Hospital and Charities Annual, 1894," and so are readily available.

The proceedings at the conference at Charing Cross Hospital on the 23rd inst. commenced by the reading of an interesting and lucid paper on the subject by the Hon. Sydney Holland. In the course of this paper Mr. Sydney Holland proposed a scheme by which the Council of the Hospital Sunday Fund should be reconstituted so as to make it representative of everybody connected with or interested in the hospitals. That is to say, subscribers, clergy, laymen, hospital managers and committee men, members of the honorary medical staff, and general practitioners were all to send representatives to this council. Out of the members of the Hospital Sunday Fund Council so constituted Mr. Holland proposes to appoint a representative Distribution Committee consisting of about fifteen members, to include five laymen, five hospital experts, four medical practitioners, and one representative of nursing. The Distribution Committee is to be charged with the duty of electing a Visiting Committee of thirty, who would visit all the metropolitan hospitals applying for a grant, and who would report to the Distribution Committee. Briefly, then, Mr. Holland's Central Board would consist of fifteen members, chosen from amongst an enlarged and strengthened Council of the Hospital Sunday Fund, which in itself would first be made representative of every interest concerned. There is much to be said, no doubt, in favour of Mr. Holland's proposals, which were received with marked attention and general acceptance by the large meeting at Charing Cross Hospital. Unfortunately, through a misunderstanding, when Mr. Holland proposed that "a Central Board for the supervision of London hospitals is needed, and that the appointment of it should no longer be delayed," he was met by an amendment, moved by Mr. Ryan, to the effect that "it is desirable to ascertain the opinion of the governing bodies of the London hospitals as to the advantage of appointing a Central Board for the supervision of London hospitals." Mr. Ryan, in the course of his speech, was careful to point out that he did not move this amendment in any opposition to the principle of a Central Board, nor did he oppose the spirit of the resolution moved by Mr. Holland, for in point of fact he highly approved of a Central Board being appointed.

The pity was that Mr. Holland's resolutions were ever separated, for the first one, which we have quoted above, was explained and limited by the second, which ran as follows: "That it is desirable to reconstitute the Hospital Sunday Fund Distribution Committee by the addition of representatives of the London hospitals, so as to form the central body representative of those associations.' Taken together these resolutions formed a concrete plan well worthy of discussion, whereas the first resolution taken by itself was a suggestion capable of the most diverse interpretation, and sure to raise opposition from those who objected to a pig in a poke, even as a gift, and much more so if it were to be paid for.

We feel it is matter for regret that the proposal of the chairman, Lord Monkswell, was not adopted, and Mr. Ryan's so-called amendment treated—as it ought to have been treated—as a rider to Mr. Holland's resolution. In the result, as will be seen from the report which appears in another column, Mr. Ryan's amendment was carried by 21 votes to 19, the majority of the persons present abstaining from voting.

If the hospitals ultimately extend anything like unanimous support to the proposals formulated by Mr. Holland, but not otherwise, the Hospital Sunday Fund Council may decide to comply with the wishes of the majority, for its members have always displayed the sincerest desire to help the hospitals to help themselves. The first step to take is to ascertain exactly how far the majority of hospital managers will go, and in what direction. If a conference can be assembled on the plan adopted in regard to the uniform system of accounts, and if such a conference appoints a committee to thresh out the subject, and in the result a memorial on behalf of the hospitals is presented by a representative deputation to the Council of the Hospital Sunday Fund, the proposal to organise a Central Hospital Board will then assume a definite and practical shape.

Hitherto there has been much discussion and great diversity of opinion, but no practical result. No doubt the question of form is of the first importance in approaching a critical question like that we are discussing. A private meeting of the representatives of all the hospitals interested should be summoned by Mr. Holland on behalf of the Hospitals Association, and as one of the most active of hospital chairmen. Their attendance at this meeting should be invited, in order to arrive at a decision as to the attitude the hospitals as a body wish to take up in regard to the Central Board. For the reasons already pointed out the proposals of the Charity Organisation Society are impracticable, and all will agree to the wisdom of attempting a settlement on the lines we have suggested. After full discussion, the meeting of hospital authorities might appoint a small committee to thresh out the details, to submit a report to an adjourned meeting, which could then appoint a deputation to put forward the decisions arrived at for the consideration of the Council of the Hospital Sunday Fund as the opinions, authoritatively expressed, of those responsible for the conduct and maintenance of the metropolitan hospitals. We shall hope to hear of such a meeting, and that Mr. Holland's proposals are receiving due consideration at the hands of the managers of all the hospitals.

